# How to Verify Plagiarism of the Paper Written in Macedonian and Translated in Foreign Language?

**DOI:** 10.3889/oamjms.2016.035

**Published:** 2016-02-29

**Authors:** Mirko Spiroski

**Affiliations:** Institute of Immunobiology and Human Genetics, Faculty of Medicine, Ss Cyril and Methodius University of Skopje, Skopje, Republic of Macedonia

**Keywords:** medical science, misconduct, plagiarism, Republic of Macedonia

## Abstract

**AIM:**

The aim of this study was to show how to verify plagiarism of the paper written in Macedonian and translated in foreign language.

**MATERIAL AND METHODS:**

Original article “Ethics in Medical Research Involving Human Subjects”, written in Macedonian, was submitted as an assay-2 for the subject Ethics and published by Ilina Stefanovska, PhD candidate from the Iustinianus Primus Faculty of Law, Ss Cyril and Methodius University of Skopje (UKIM), Skopje, Republic of Macedonia in Fabruary, 2013. Suspected article for plagiarism was published by Prof. Dr. Gordana Panova from the Faculty of Medical Sciences, University Goce Delchev, Shtip, Republic of Macedonia in English with the identical title and identical content in International scientific on-line journal “SCIENCE & TECHNOLOGIES”, Publisher “Union of Scientists - Stara Zagora”.

**RESULTS:**

Original document (written in Macedonian) was translated with Google Translator; suspected article (published in English pdf file) was converted into Word document, and compared both documents with several programs for plagiarism detection. It was found that both documents are identical in 71%, 78% and 82%, respectively, depending on the computer program used for plagiarism detection. It was obvious that original paper was entirely plagiarised by Prof. Dr. Gordana Panova, including six references from the original paper.

**CONCLUSION:**

Plagiarism of the original papers written in Macedonian and translated in other languages can be verified after computerised translation in other languages. Later on, original and translated documents can be compared with available software for plagiarism detection.

## Introduction

Plagiarism of the papers is one of the biggest problems in publication ethics worldwide. Simple definition of plagiarism given in the Merriam-Webster Dictionary is: the act of using another person’s words or ideas without giving credit to that person or the act of plagiarizing something [1].

Committee on Publication Ethics (COPE) provides advice to editors and publishers on all aspects of publication ethics and, in particular, how to handle cases of research and publication misconduct. It also provides a forum for its members to discuss individual cases. COPE does not investigate individual cases but encourages editors to ensure that cases are investigated by the appropriate authorities (usually a research institution or employer) [2].

COPE published flowcharts on what to do if you suspect plagiarism: (a) suspected plagiarism in a submitted manuscript [3] and (b) suspected plagiarism in a published manuscript [4].

If plagiarism was documented, then retraction of the plagiarised paper is mandatory. Retraction is a mechanism for correcting the literature and alerting readers to publications that contain such seriously flawed or erroneous data that their findings and conclusions cannot be relied upon. Unreliable data may result from honest error or from research misconduct. Retractions are also used to alert readers to cases of redundant publication (i.e. when authors present the same data in several publications), plagiarism, and failure to disclose a major competing interest likely to influence interpretations or recommendations. The main purpose of retractions is to correct the literature and ensure its integrity rather than to punish authors who misbehave [5].

System for plagiarism detection and analysis was developed and installed from Ministry of Education and Science, Republic of Macedonia. This system provides an easy and efficient way of detecting documents that contain plagiarised parts from already published and presented documents. The system’s goal is to provide an easy and intuitive interface for uploading documents. The students and researchers/scientists can easily upload their homework, bachelor’s thesis, master’s thesis, doctoral thesis (dissertation) and other published papers and documents. The system also provides a mechanism for comparing the uploaded documents with all the other documents that are already present in the system and to measure their originality, i.e. to detect if any of the content is already published [6]. Unfortunately the system is robust, language restricted, and closed database is limited to the documents deposited in the local website only.

The aim of this study was to show how to verify plagiarism of the paper written in Macedonian and translated in English or other languages.

## Material and Methods

Original article “Ethics in Medical Research Involving Human Subjects”, written in Macedonian, was submitted as an assay-2 for the subject Ethics and published by Ilina Stefanovska, PhD candidate from the Iustinianus Primus Faculty of Law, Ss Cyril and Methodius University of Skopje (UKIM), Skopje, Republic of Macedonia in Fabruary, 2013. The assay was published and is publicly available at the website of Institute of Immunobiology and Human Genetics at the Faculty of Medicine, UKIM, Skopje, Republic of Macedonia [7].

**Figure 1:**
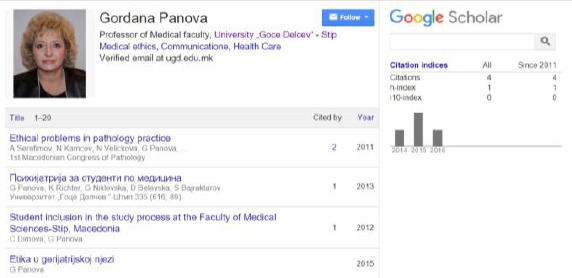
*Google Scholar user profile of Prof. Dr. Gordana Panova from the Faculty of Medical Sciences, University Goce Delchev, Shtip, Republic of Macedonia*.

Two years later (in 2015 year) Prof. Dr. Gordana Panova from the Faculty of Medical Sciences, University Goce Delchev, Shtip, Republic of Macedonia (Figure 1) published a paper in English with the identical title and identical content in International scientific on-line journal “SCIENCE & TECHNOLOGIES”, Publisher “Union of Scientists - Stara Zagora” [8, 9].

In order to compare two papers and verify the plagiarism, the original paper written in Macedonian [7] was translated in English by Google translator. Plagiarised paper, published in English [9], was converted to Word document. And finally, both Word document were compared with several softwares for checking plagiarism (Copyscape [10], WCopyfind [11], and Plagiarism Checker [12]).

**Figure 2:**
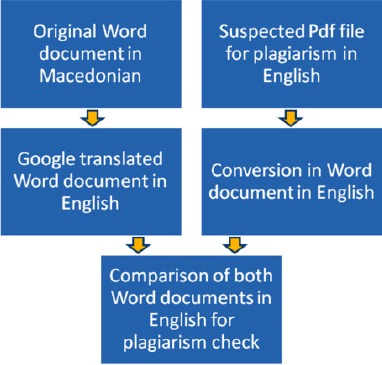
*Several steps for checking plagiarism of the paper written in Macedonian and suspected paper translated in English*.

## Results

**Figure 3:**
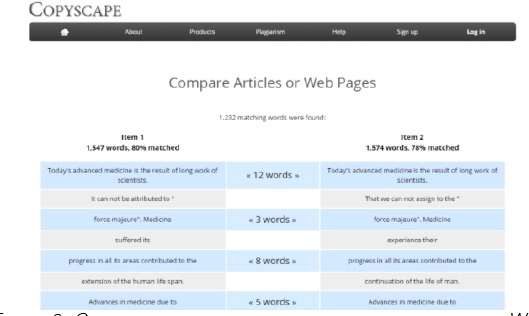
*Comparison of original and suspected articles as a Word document with the Copyscape program. Suspected paper (Item 2, on the right) contains 1574 words and was 78% matched*.

An original and suspected paper were compared with Copyscape program and was found that they have almost identical number of words (1547 words in the original paper and 1574 words in suspected paper or Item 2). It was found that 1232 words were matched or 78% of the words were matched (Fig. 3).

**Figure 4:**
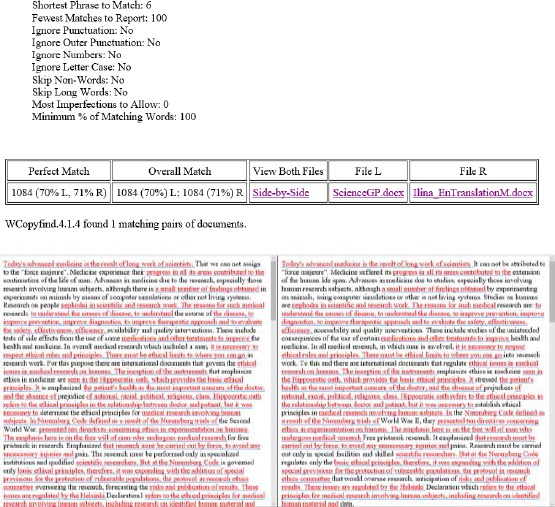
*Comparison of original and suspected articles as a Word document with the WCopyfind program. Overall match (labelled with red) was 71% between original and suspected paper*.

Comparison of the two papers with the WCopyfind program has shown that overall match was 71% between original and suspected paper. Identical sentences in both documents were labelled with red (Fig. 4).

**Figure 5:**
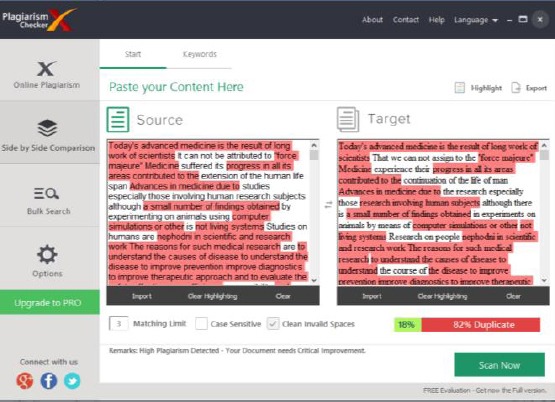
*Comparison of original and suspected articles as a Word document with the Plagiarism Checker program. Target (suspected) document was 82% duplicate with the Source (original) document*.

The results of the comparison of the original and suspected papers analyzed with Plagiarism Checker program are shown in Fig. 5. Target (suspected) document was 82% duplicate with the Source (original) document. Identical sentences are labeled in red.

## Discussion

In this paper I presented how to verify plagiarism of document written in Macedonian and translated in English language. I translated original document written in Macedonian with Google Translator, converted plagiarised pdf file into Word document, and compared both documents with several programs for plagiarism detection. I found that both documents are identical in 71%, 78% and 82%, respectively, depending on the computer program I used for plagiarism detection.

It was obvious that original paper [7] was entirely plagiarised by Prof. Dr. Gordana Panova [9], including six references from the original paper. This is not isolated case, but rather serial case of plagiarism of Prof. Dr. Gordana Panova. Four retractions in Društvo defektologa Vojvodine, Novi Sad, Serbia from the same author were published [13, 14] and were reported in Retraction Watch [15].

Common characteristics of four retracted papers [16-19] from Prof. Dr. Gordana Panova are: original papers are parts or entire texts of students; original papers are written in Macedonian; plagiarised papers are translated in English; plagiarised papers are presented at the conferences outside Republic of Macedonia; plagiarised papers are published as a Book of Abstracts or Book of Papers without reviews (conference papers).

Research and publication misconduct may not be an isolated incident. In many cases, when serious misconduct comes to light, investigation of the researcher’s earlier work reveals further problems. Therefore, when a researcher is found to have committed serious misconduct (such as data fabrication, falsification or plagiarism) the institution should review all the individual’s publications, including those published before the proven misconduct took place. In such cases, it may be necessary to alert previous employers to enable them to review work carried out by the discredited researcher when working at their institution, to determine the reliability of publications arising from that work [20].

After this verification of plagiarised paper by Prof. Dr. Gordana Panova, I expect two appropriate reactions, one from the Editor-in-Chief of the International scientific on-line journal “SCIENCE & TECHNOLOGIES”, Publisher “Union of Scientists - Stara Zagora”, and another one from the Dean of the Faculty of Medical Sciences, University Goce Delchev, Shtip, Republic of Macedonia.

Editor-in-Chief of the International scientific on-line journal “SCIENCE & TECHNOLOGIES” should follow the Retraction guidelines from the Committee on Publication Ethics [5]. In short, he/she will publish a letter for retraction of the plagiarized paper [for examples of this see reference 21] and label all pages of the published paper with the watermark “Retracted” [for examples of this see reference 22].

Dean of the Faculty of Medical Sciences, University Goce Delchev, Shtip, Republic of Macedonia should create Independent Body of experienced professors, from the fields of medicine and ethics, with the task to analyse all published papers of Prof. Dr. Gordana Panova for possible plagiarism and/or other misconducts [3-5, 20].

This comparison of the original paper written in Macedonian with suspected paper translated into foreign languages can be modified with back translation. Namely, suspected paper written in any language can be translated and compared with the original paper written in the identical language. I suppose that by using these methods, detection of plagiarized papers in Macedonia will be possible and percentage of plagiarized papers will increase very much. It is also necessary to support programmers to implement these possibilities of translation and back translations into the actual computer programs fro plagiarism detection.

In conclusion, plagiarism of the original papers written in Macedonian and translated in other languages can be verified after computerised translation in other languages. Later on, original and translated documents can be compared with available software for plagiarism detection.
